# The FLT3-ITD mutation and the expression of its downstream signaling intermediates STAT5 and Pim-1 are positively correlated with CXCR4 expression in patients with acute myeloid leukemia

**DOI:** 10.1038/s41598-019-48687-z

**Published:** 2019-08-21

**Authors:** Tingyong Cao, Nenggang Jiang, Hongyan Liao, Xiao Shuai, Jun Su, Qin Zheng

**Affiliations:** 1 Department of Hematology, West China Hospital of Sichuan University, Chengdu, 610041 China; 2 Department of Laboratory Medicine, West China Hospital of Sichuan University, Chengdu, 610041 China

**Keywords:** Leukaemia, Acute myeloid leukaemia

## Abstract

Chemokine ligand 12(CXCL12) mediates signaling through chemokine receptor 4(CXCR4), which is essential for the homing and maintenance of Hematopoietic stem cells (HSCs) in the bone marrow. FLT3-ITD mutations enhance cell migration toward CXCL12, providing a drug resistance mechanism underlying the poor effects of FLT3-ITD antagonists. However, the mechanism by which FLT3-ITD mutations regulate the CXCL12/CXCR4 axis remains unclear. We analyzed the relationship between CXCR4 expression and the FLT3-ITD mutation in 466 patients with *de novo* AML to clarify the effect of FLT3-ITD mutations on CXCR4 expression in patients with AML. Our results indicated a positive correlation between the FLT3-ITD mutant-type allelic ratio (FLT3-ITD MR) and the relative fluorescence intensity (RFI) of CXCR4 expression in patients with AML (r = 0.588, P ≤ 0.0001). Moreover, the levels of phospho(p)-STAT5, Pim-1 and CXCR4 proteins were positively correlated with the FLT3-ITD MR, and the mRNA levels of CXCR4 and Pim-1 which has been revealed as one of the first known target genes of STAT5, were upregulated with an increasing FLT3-ITD MR(P < 0.05). Therefore, FLT3-ITD mutations upregulate the expression of CXCR4 in patients with AML, and the downstream signaling intermediates STAT5 and Pim-1 are also involved in this phenomenon and subsequently contribute to chemotherapy resistance and disease relapse in patients with AML. However, the mechanism must be confirmed in further experiments. The combination of CXCR4 antagonists and FLT3 inhibitors may improve the sensitivity of AML cells to chemotherapy and overcome drug resistance.

## Introduction

Acute myeloid leukemia (AML) is a genetically heterogeneous and malignant clonal disorder of hematopoietic stem cells (HSCs) characterized by the proliferation of abnormal myeloid progenitors and impaired differentiation into mature cells^1^. The treatment and prognosis of AML patients depend on accurate cytogenetic and genetic examinations^2^. FMS-like tyrosine kinase 3-internal tandem duplication (FLT3-ITD) mutations are the most frequently identified genetic alterations in AML and are detected in approximately 20–25% of AML patients^3,4^. FLT3-ITD has been demonstrated to be significantly associated with a higher relapse rate and inferior overall survival (OS) in AML. Furthermore, the response to salvage therapy in relapsed FLT3-ITD-mutated AML is also consistently poor^5^. Recent data suggest that a high FLT3 mutant-type allelic ratio (FLT3-ITD MR) is associated with an increased risk of relapse and reduced OS^6,7^. Although several FLT3-ITD antagonists have been developed, due to drug resistance, few are effective for the treatment of AML with FLT3-ITD mutations^8^. The mechanisms responsible for drug resistance include the acquisition of additional mutations in the FLT3 gene and/or the activation of other prosurvival pathways such as microenvironment-mediated resistance^9,10^. Chemokine ligand 12 (CXCL12), also known as stromal-derived factor 1 (SDF-1α), is highly expressed in the bone marrow niche. Signals from the CXCL12-chemokine receptor 4 (CXCR4) axis have been shown to act as critical mediators in interactions between leukemia cells and the microenvironment, which are the major cause of chemotherapy resistance and disease relapse in AML^11,12^. Although some studies suggest that FLT3-ITD mutations enhance AML cell migration toward CXCL12^13,14^, the mechanism by which FLT3-ITD mutations regulate the CXCL12/CXCR4 axis is unknown. Clarification of this mechanism is essential for the development of an efficient combined therapeutic approach.

FLT3-ITD mutation-mediated signaling transduction has been characterized as the aberrant expression and constitutive activation of STAT5^15^. Pim-1, an oncogenic serine/threonine kinase, has been revealed as one of the first known target genes of STAT5^16^ and was also found to be essential for CXCR4 surface expression and intracellular receptor processing^17^. Therefore, we hypothesize that FLT3-ITD can induce CXCR4 expression through the downstream STAT5 signaling pathway, in which Pim-1 acts as a central regulator. CXCR4 antagonists can thus disrupt the migration of FLT3-ITD-mutated AML cells toward CXCL12. We further reason that combining CXCR4 antagonists with FLT3 inhibitors can sensitize AML cells to chemotherapy and overcome drug resistance. The purpose of this study is to examine the expression of FLT3-ITD, STAT5/p-STAT5, Pim-1, and CXCR4 in experimental and control groups and to elucidate the importance of the CXCL12/CXCR4 axis to the pathogenesis of FLT3-ITD-mutated AML.

## Materials and Methods

### Patients and controls

Four hundred sixty-six patients (250 females and 216 males) with *de novo* AML before treatment and 20 healthy controls (11 females and 9 males) were randomly recruited from the West China Hospital of Sichuan University between September 2011 and November 2015 [This patient cohort was also used in our previously published study (Zheng *et al*. (2016) Gene, 588:103) designed to determine the associations between the polymorphisms in the SDF-1 (rs1801157, GNA) and CXCR4 (rs2228014, CNT) genes with susceptibility and leukemia cell dissemination in AML, a completely different goal from the present study]. AML cases were classified according to the French-American-British (FAB) committee recommendations and World Health Organization (WHO)^18,19^ criteria by a combination of clinical, morphological, immunophenotypic, and genetic features. All bone marrow samples from AML patients were obtained at the time of diagnosis. Mononuclear cells were isolated by Ficoll separation solution (GE Healthcare, USA), bone marrow samples smears were used to evaluate the primary AML cell, typically resulting in a population containing more than 95% blasts. For a few samples with insufficient primary AML cell, flow cytometry is used for sorting. After isolation, cells were subjected to controlled freezing and stored in liquid nitrogen.

MV4-11 (human acute myeloid monocytic leukemia) cells were used as the FLT3-ITD-mutated cell line; HL-60 (human acute promyelocytic leukemia, APL) cells were used as the FLT3-wild-type (FLT3-wt) cell line. Both the MV4-11 and HL-60 cells were obtained from the Hematology Disease Research Laboratory of the West China Hospital of Sichuan University.

### CXCR4 expression analysis by flow cytometry

Single-cell suspensions of the studied cells were adjusted to a concentration of 1 × 10^6^ cells/ml. Red blood cells were lysed before analysis. Twenty-microliter volumes of cell suspensions were incubated with 20 μl of an APC-conjugated mouse anti-human anti-CXCR4 antibody (CD184-APC, BD Pharmingen, USA) in combination with anti-CD34-FITC, anti-CD117-PE, and anti-CD45-PerCP antibodies (Becton Dickinson, USA). Nonspecific binding was assessed by an APC-conjugated mouse anti-human IgG2, κ (Becton Dickinson, USA) antibody as an isotype control. The cell suspensions were analyzed by flow cytometry (BD FACS Canto II, Becton Dickinson, USA). The results are shown as the relative fluorescence intensity (RFI) values. The RFI of CXCR4 expression on the surface of leukemia cells or on CD34^+^ cells in healthy controls was calculated by dividing the fluorescence intensity of anti-CXCR4-APC expression by that of isotype-APC expression.

### Polymerase chain reaction (PCR) for the amplification of exons 14 and 15

Genomic DNA was extracted from 200 μl of prepared mononuclear cell suspension using a QIAamp DNA Blood Mini Kit (Qiagen, Germany) according to the manufacturer’s protocol. PCR was performed on genomic DNA using published primer molecules^20^. The size of the target gene fragment was 329 bp. Briefly, 2 µl of DNA was amplified in a volume of 50 µl containing 1 µl of the forward primer (14F-5′-GCAATTTAGGTATGAAAGCCAGC-3′) (Invitrogen, USA) (10 μg/μl), 1 µl of the reverse primer (15R-5′-CTTTCAGCATTTTGACGGCAACC-3′) (Invitrogen, USA) (10 μg/μl), 2 µl of Taq DNA polymerase (5 U/μl), 4 µl of dNTPs, 5 µl of 10× buffer and 35 µl of H_2_O (Takara, Japan). The thermal cycling conditions were as follows: initial denaturation at 94 °C for 150 s, followed by 35 cycles of denaturation at 94 °C for 30 s, annealing at 57 °C for 30 s, and extension at 72 °C for 2 min. After amplification, the PCR products underwent a final annealing step at 72 °C for 7 min, followed by cooling at 4 °C for 10 min. The products were subjected to 2% agarose gel electrophoresis using appropriate size markers to verify the size of the PCR products.

### GeneScan analysis of the FLT3-ITD MR

The PCR products were purified by an Agarose Gel DNA Purification Kit (Takara, Japan). For GeneScan analysis, the 5′ end of the FLT3 14 F PCR primer was labeled with FAM (TIB MOLBIOL, Germany). The purified amplified products were mixed with the GeneScan 500 LIZ™ Size Standard internal lane standard (Life Technologies, USA) and highly deionized formamide (Applied Biosystems, USA) (1:4:40). After vigorous shaking, the reaction system was heated to 95 °C for 5 min and then rapidly placed on ice for 5 min. An ABI 3730XL DNA Analyzer (Applied Biosystems, USA) was used for GeneScan analysis. In capillary electrophoresis, FLT3-wt samples produced a single peak at 329 bp, while additional peaks corresponding to sizes greater than 329 bp indicated the presence of FLT3-ITDs. The FLT3-ITD MR was calculated according to the following formula: mutant peak area/mutant peak area + wild-type peak area.

### Sequencing

To further verify the presence of FLT3-ITD mutations, the purified PCR products were cycle sequenced using a BigDye® Terminator version 3.1 Cycle Sequencing Kit (Applied Biosystems, USA) on the ABI 3730XL DNA Analyzer (Applied Biosystems, USA), according to the manufacturer’s protocol.

### Quantitative real-time PCR (RT-PCR)

RNA was prepared from sorted cells using a QIAamp RNA Blood Mini Kit (QIAGEN, Germany) according to the manufacturer’s protocol and was reverse transcribed to cDNA using a PrimeScript™ RT reagent kit (Takara, Japan). PCR was performed using SYBR® Green PCR Master Mix (Applied Biosystems, USA) by a Roche LightCycler® 480 Real-Time PCR System (Roche Diagnostics, Germany). The following oligonucleotides were used as PCR primers: *h*CXCR4 (76-bp amplicon): 5′-ATGAAGGAACCCTGTTCCCGT-3′ (forward) and 5′-AGATGATGGAGTAGATGGTGGG-3′ (reverse); *h*Pim-1 (119-bp amplicon): 5′-CGAGCATGACGAAGAGATCAT-3′ (forward) and 5′-TCGAAGGTTGGCCTATCTGA-3′ (reverse); and *h*GAPDH (149-bp amplicon): 5′-GTGGTCTCCCTGACTTTCAACAGC-3′ (forward) and 5′-ATGAGGTCCACCACCTGCTTGCTG-3′ (reverse). The thermal cycling parameters were as follows: 40 cycles at 94 °C for 30 s, 58 °C for 30 s and 72 °C for 30 s. Expression levels were normalized to those of GAPDH using the Ct method.

### ***In vitro*** proliferation assay

The MV4-11 and HL-60 cell lines were divided into three groups: (i) Blank control group: cells incubated in pure medium; (ii) Negative control group: cells incubated without drugs; (iii) Drug experimental group: cells incubated with sorafenib, AMD3100, or sorafenib in combination with AMD3100. The leukemia cells were treated with different doses of sorafenib (20 nM, 100 nM, 200 nM, 400 nM, and 1 μM), AMD3100 (500 ng/ml, 1 μg/ml, 5 μg/ml, and 10 μg/ml), and sorafenib in combination with AMD3100.

MV4-11 and HL-60 cell suspensions were seeded in 96-well plates (1 × 10^5^ cells/ml, 100 μl per well), and the plates were incubated in a humidified incubator at 37 °C for 24 h, 48 h and 72 h. For cell proliferation analysis, a Cell Counting Kit-8 (DOJINDO, Japan) was used according to the manufacturer’s instructions. The absorbance (optical density, OD) was measured at 450 nm using a microplate reader (Thermo Scientific, USA). The experiments were repeated three times. The following equation was used to calculate the cell proliferation inhibition rate: Cell proliferation inhibition rate (IR, %) = 1 − ((experimental group OD − blank control group OD)/(negative control group OD − blank control group OD)) × 100%.

### Apoptosis analysis

For cell apoptosis assays, MV4-11 or HL-60 cells were treated with various concentrations of AMD-3100 (500 ng/ml, 1 μg/ml, 5 μg/ml, and 10 μg/ml) or sorafenib (100 nM, 400 nM, 1 μM, and 5 μM) for 24 h, 48 h, and 72 h. The number of drug-induced apoptotic cells was quantified by determining the number of annexin V-positive cells using an Annexin V Apoptosis Detection Kit(BD Pharmingen, USA). The experiments were repeated three times.

### Cell chemotaxis assays

A total of 90 μl of MV4-11 and HL-60 cell suspensions were incubated with 10 μl of AMD3100 at different concentrations (500 ng/ml, 1 μg/ml, 5 μg/ml, and 10 μg/ml) in the upper chambers of Transwell plates (24-Well Millicell Hanging Cell Culture Inserts, Costar Corning, USA) with a diameter of 6.5 mm and a pore size of 8.0 μm. Medium containing 100 nM *CXCL12*(R&D Systems, USA) was added to the lower chambers of the Transwell plates. The Transwell plates were then incubated for 4 h at 37 °C and 10% CO_2_. After incubation, the number of cells that had migrated to the lower chamber was detected using a Sysmex XN-5000 automated hematology analyzer (Sysmex, Japan). A 90-μl volume of the MV4-11 and/or HL-60 cell suspensions and 10 μl of PBS were added to the upper chambers of Transwell plates as the negative control groups. The chemotaxis rate was calculated by dividing the number of seeded cells by the number of migrated cells and subtracting the number of cells that migrated toward untreated medium. The experiments were repeated three times.

### Western blot analysis

The protein expression levels of CXCR4, Pim-1, and STAT5/p-STAT5 in the experimental and control groups and the expression levels of p38 mitogen-activated protein kinase (MAPK)/p-p38 MAPK in MV4-11 and HL-60 cells incubated with different concentrations of AMD3100 (500 ng/ml, 1 μg/ml, 5 μg/ml, and 10 μg/ml) for 48 h were analyzed by Western blotting using standardized protocols. The following antibodies were used: anti-CXCR4 (BD Pharmingen, USA), anti-Pim-1 (Cell Signaling, USA), anti-STAT5 (Abcam, UK), anti-phospho (p)-STAT5 (Y694) (Abcam, UK), anti-p38 MAPK (Abcam, UK) and anti-p-p38 MAPK (Thr180/Tyr182) monoclonal antibodies (Abcam, UK).

### Statistical analysis

Statistical analysis was performed using SPSS Statistics 24.0 software (Chicago, IL, USA). Comparisons of continuous variables between two groups were carried out using the Mann-Whitney U test. One-way ANOVA was used to examine the differences among different groups. The Kruskal-Wallis H test was used to analyze comparisons among more than two groups. Pearson correlation analysis was applied to analyze the relationship between different variables. Two-sided P values of < 0.05 were considered statistically significant. GraphPad Prism 6.0 (GraphPad Software, San Diego, CA) was used as the plotting tool.

## Results

### Clinical and laboratory characteristics of patients in the AML experimental group and individuals in the healthy control group

Four hundred sixty-six patients with *de novo* AML were recruited before treatment. In terms of AML subtypes, the AML group comprised 84 patients with M1, 173 patients with M2, 51 patients with APL (M3), 64 patients with M4, 67 patients with M5 and 27 patients with M6. The age of the patients ranged from 14 to 89 years, with a median of 48 years. The baseline characteristics of the participants with AML are described in Table 1. Twenty healthy donors with a median age of 48 years comprised the healthy control group.

**Table 1:** Clinical and laboratory characteristics of the *de novo* AML patients.

FAB (n)	WBC (109/L) median, range	Hb (g/L) median, range	PLT (109/L) median, range	BMLC (%) median, range	PBLC (109/L) median, range	FLT3-ITD N* (%)	FLT3- ITD MR* median, range	CXCR4 RFI^#^ Median ± s
Total (466)	8.82 (0.39–496.84)	77 (32–162)	34 (3–504)	70.0 (8.5–97.5)	3.09 (0–343.01)	101 (21.67%)	0.32 (0.03–0.91)	6.31 ± 6.01
M1 (84)	8.48 (0.47–326.87)	78 (38–160)	30 (5–365)	92 (26–97.5)	4.93 (0–320.33)	15 (17.86%)	0.37 (0.07–0.73)	6.82 ± 7.17
M2 (173)	7.82 (0.39–496.84)	76 (32–162)	32 (5–385)	44.5 (13.5–86.5)	1.53 (0–198.74)	27 (15.61%)	0.31 (0.04–0.89)	5.92 ± 4.93
M3 (51)	3.17 (0.8–115.82)	78 (43–121)	26 (3–134)	77.8 (40–96)	0.53 (0–92.96)	18 (35.29%)	0.33 (0.03–0.59)	6.29 ± 5.83
M4 (64)	26.26 (0.69–368.1)	77 (34–134)	43 (6–480)	76 (22.5–96.5)	12.7 (0–343.01)	15 (23.44%)	0.18 (0.03–0.91)	9.70 ± 8.20
M5 (67)	22.19 (0.45–372.3)	79 (37–144)	40 (2–478)	77.2 (21.5–98)	11.07 (0–327.08)	24 (35.82%)	0.33 (0.03–0.83)	8.72 ± 5.30
M6 (27)	2.45 (1.02–20.33)	70 (38–127)	48 (7–504)	14 (8.5–40)	0.07 (0–7.73)	2 (7.41)	0.22 (0.18–0.27)	4.76 ± 5.27

**Figure 1:**
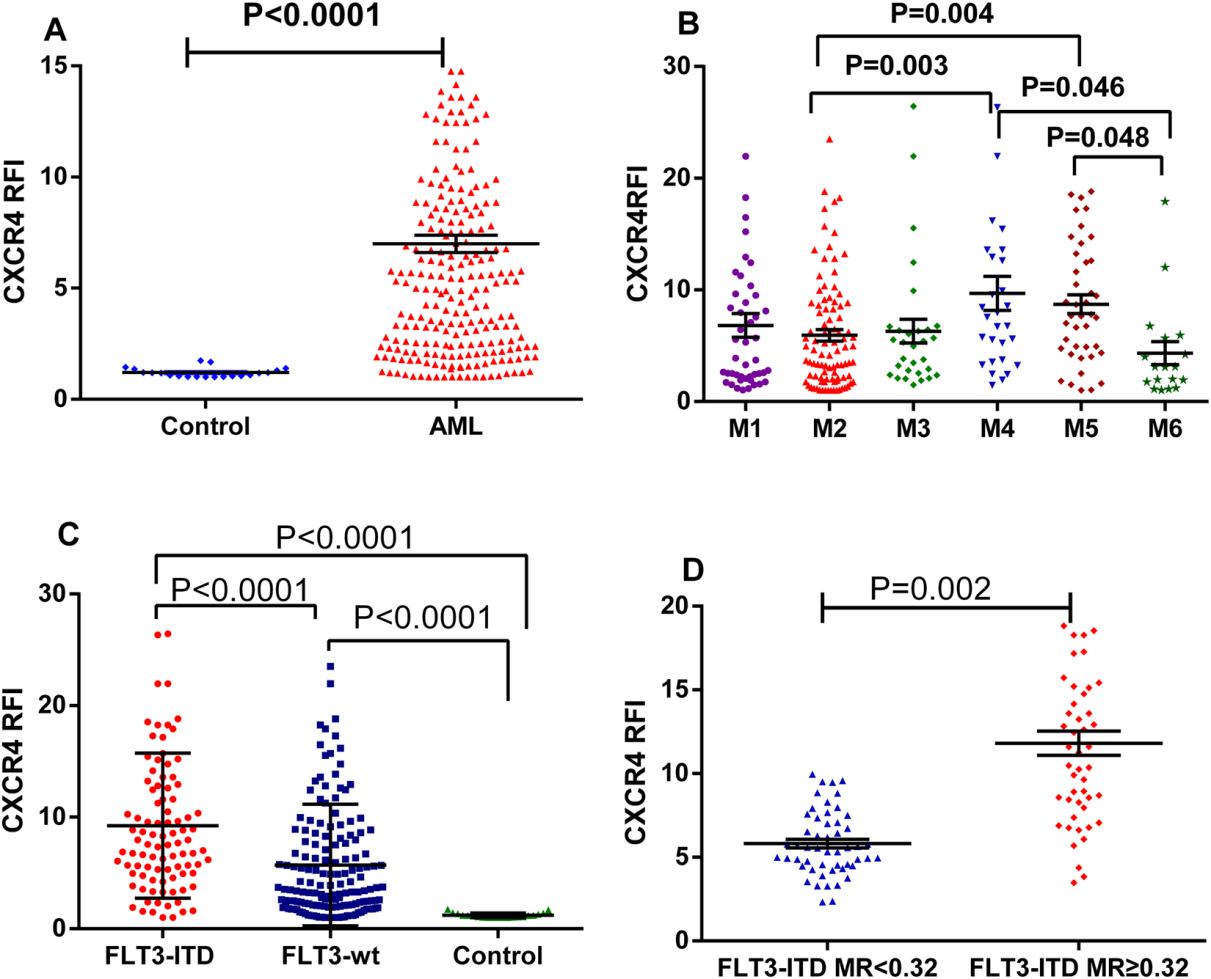
Distributions of the RFI values for CXCR4 expression in control individuals and patients with AML. The distributions of the RFI values for CXCR4 expression in controls and patients with AML are presented as a scatter plot, the S.E.M is shown as a “T”, and the median RFI of CXCR4 expression in each group is shown as a horizontal line. (**A**) Comparison of the RFI values for CXCR4 expression between the patients with AML(n = 466) and the control group(n = 20). (**B**) Comparison of the CXCR4 RFI values among patients with AMLstratified by FAB subtype (M1 n = 84, M2 n = 173, M3 n = 51, M4 n = 64, M5 n = 67, and M6 n = 27). (**C**) Comparison of the CXCR4 RFI values among the FLT3-ITD mutation (n = 101), FLT-wt (n = 365) and control groups (n = 20). (**D)** Comparison of the CXCR4 RFI values between the FLT3-ITD MR ≥ 0.32(n = 51) and FLT3-ITD MR < 0.32 groups (n = 50).

WBC: White Blood Cell Count: Hb: Hemoglobin Level: PLT: Platelet Count: BMLC: Bone Marrow leukemia cell Percentage: PBLC: Peripheral Blood Leukemia cell Count; FLT3-ITD MR: FLT3-ITD mutant type allelic ratio; CXCR4 RFI: relative fluorescence intensity (RFI) of CXCR4. *The difference of FLT3-ITD MR between the FAB subgroups was not statistically significant (p = 0.864). ^#^The difference of CXCR4 RFI between the FAB subgroups was seen in Fig. 1B.

### FLT3-ITD mutations and CXCR4 expression in the AML experimental group

Of the 466 patients with AML, 101 (21.67%) had FLT3-ITD mutations. The length of the inserted genes in the FLT3-ITD mutations was 3~108 bp, with an average length of 42 bp. FLT3-ITD mutations were detected in patients with all subtypes of AML. The highest prevalence of FLT3-ITD mutations was 35.82% and occurred in the AML-M5 subtype, followed by 35.29% in the APL subtype. The lowest prevalence was 7.41% and occurred in the AML-M6 subtype (Table 1). The FLT3-ITD MR in the AML group ranged from 0.03 to 0.91, with a median of 0.32 (Table 1). No significant difference was observed in the FLT3-ITD MR among the subgroups (P = 0.864).

The RFI of CXCR4 expression on bone marrow leukemia cells was 6.31 ± 6.01 (median ± SD), as assessed using flow cytometry, and the value was significantly higher than control cells (1.19 ± 0.21, P ≤ 0.0001, Fig. 1A), indicating that CXCR4 expression was upregulated. Pearson’s correlation analysis did not reveal correlations between the CXCR4 RFI on the surface of AML cells and the peripheral white blood cell (WBC) count, platelet (PLT) count, hemoglobin (HGB) concentration, peripheral blood leukemic cell (PBLC) count or the proportion of leukemia cells in the bone marrow (all P > 0.05, details are shown in Table 2). Comparisons within the subgroups revealed that the CXCR4 RFI was ranked as M4 > M5 > M1 > M3 > M2 > M6, and the M4 and M5 subgroups exhibited significantly upregulated CXCR4 expression compared to that in the M2 and M6 subgroups (P = 0.003, P = 0.004, P = 0.046, and P = 0.048, respectively, Fig. 1B).

**Table 2:** Pearson Correlation between CXCR4 RFI and laboratory characteristics in AML group.

Parameter(n)	Median, range	*r* ^#^	*P*
CXCR4 RFI	6.31 (0.74–40.17)		
FLT3-ITD MR	0.32 (0.03–0.91)	0.588	***0.000***
WBC	8.82 (0.39–496.84)	0.071	0.361
PLT	34 (3–504)	0.043	0.666
Hb	77 (32–162)	0.089	0.374
PBLC	3.09 (0–343.01)	0.013	0.870
BMLC	70.0 (8.5–97.5)	0.006	0.934

CXCR4 RFI: relative fluorescence intensity (RFI) of CXCR4; FLT3-ITD MR: FLT3-ITD mutant type allelic ratio; WBC: White Blood Cell Count: PLT: Platelet Count: Hb: Hemoglobin Level: PBLC: Peripheral Blood Leukemia cell Count; BMLC: Bone Marrow leukemia cell Percentage.

### FLT3-ITD mutations were associated with elevated CXCR4 expression on the leukemic cell surface in the AML group

A significantly higher CXCR4 RFI was detected on the surface of leukemia cells in the FLT3-ITD mutated AML group (9.24 ± 5.51, mean ± SD) than in the FLT3-wt group (5.72 ± 4.44, P ≤ 0.0001, Fig. 1C). We then categorized patients with AML carrying the FLT3-ITD mutations into two subgroups according to the median FLT3-ITD MR value of 0.32. The subgroup with an FLT3-ITD MR of ≥ 0.32 (11.90 ± 6.02) showed a significantly higher CXCR4 RFI than that the subgroup with an FLT3-ITD MR of < 0.32 (7.73 ± 6.33, P = 0.002, Fig. 1D). We also identified a positive correlation between the FLT3-ITD MR and the CXCR4 RFI on the surface of leukemia cells (r = 0.588, P ≤ 0.0001, Table 2).

### Comparison of STAT5/p-STAT5, Pim-1 and CXCR4 levels

**Figure 2:**
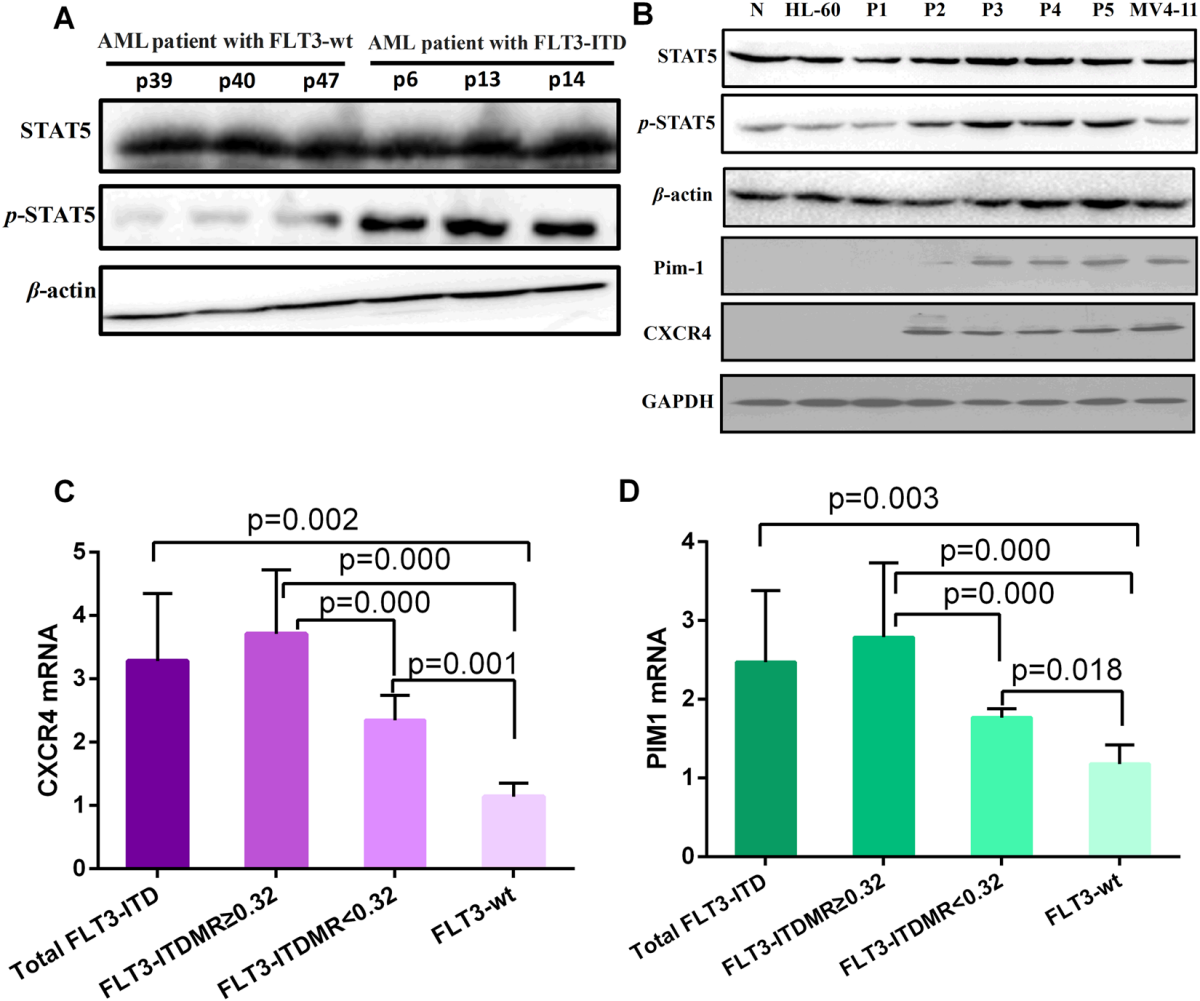
Comparison of the levels of STAT5/p-STAT5, Pim-1 and CXCR4 proteins expression levels between the FLT3-ITD mutation group and the FLT3-wt group. (**A**) Levels of the STAT5/p-STAT5 proteins in patient with AML carrying FLT3-wt (patients 39, 40, and 47) or FLT3-ITD mutations (patients 6, 13, and 14). A significantly higher p-STAT5 level was detected in patients with AML carrying FLT3-ITD mutations than in patients with AML carrying FLT3-wt. (**B**) Levels of the STAT5/p-STAT5, Pim-1 and CXCR4 proteins. N: Normal control; HL-60: AML standard cell line expressing FLT3-wt; P1: patient with AML carrying FLT3-wt; P2: patient with AML and an FLT3-ITD MR = 0.20; P3: patient with AML and an FLT3-ITD MR = 0.32; P4: patient with AML and an FLT3-ITD MR = 0.45; P5: patient with AML and an FLT3-ITD MR = 0.76; MV4-11: AML standard cell line with FLT3-ITD mutations. (**C**,**D**) The comparison of CXCR4 and Pim-1 mRNA expression levels among the FLT-wt (n = 50), FLT3-ITD MR ≥ 0.32 (n = 51) and FLT3-ITD MR < 0.32 (n = 50) groups is presented as a histogram (all P < 0.05). The median value of each group is represented by the height of the bar, and the error bars are shown as a “T” above each bar in the histogram.

We compared the protein expression level of p-STAT5/STAT5, Pim-1 and CXCR4 among the FLT3-ITD-mutated, FLT3-wt and normal control groups using Western blotting to further examine whether CXCR4 expression in leukemia cells is regulated by FLT3-ITD mutations. First, significantly higher levels of p-STAT5 were detected in patients with AML carrying FLT3-ITD mutations than in patients with AML carrying FLT3-wt (Fig. 2A). The level of the p-STAT5 protein gradually increased as the FLT3-ITD MR increased in the FLT3-ITD mutated group, in contrast to the normal control group, which exhibited fairly low p-STAT5 levels. Furthermore, levels of the Pim-1 and CXCR4 proteins expression were detected in patients with AML carrying FLT3-ITD mutants and MV4-11 cells but not in the normal control group, patients with AML carrying FLT3-wt or HL-60 cells (Fig. 2B).

Then, we compared the expression of the Pim-1 and CXCR4 mRNAs between the FLT3-ITD MR ≥ 0.32 subgroup, FLT3-ITD MR < 0.32 subgroup and FLT3-wt subgroup using RT-PCR. First, the levels of the Pim-1 and CXCR4 mRNAs were 2.47 ± 0.91 and 3.28 ± 1.07 (mean ± SD) in the FLT3-ITD group, respectively, values that were significantly higher than in the FLT3-wt group (1.18 ± 0.24 for the Pim-1 mRNA and 1.14 ± 0.21 for the CXCR4 mRNA, respectively; P = 0.003 and P = 0.002 for Pim-1 and CXCR4, respectively, Fig. 2C,D). Furthermore, positive correlations were observed between the FLT3-ITD MR and the levels of the CXCR4 and Pim-1 mRNAs in leukemia cells using the Pearson correlation analysis (r = 0.693, P ≤ 0.0001; r = 0.587, P ≤ 0.0001, respectively), and between the expression of the CXCR4 mRNA and Pim-1 mRNA (r = 0.726, P ≤ 0.0001). Moreover, the levels of the CXCR4 mRNA and Pim-1 mRNA were significantly different between the FLT3-wt, FLT3-ITD MR < 0.32 and FLT3-ITD MR ≥ 0.32 subgroups (P ≤ 0.0001 and P ≤ 0.0001 for CXCR4 and Pim-1, respectively). The level of the CXCR4 mRNA in the FLT3-ITD MR ≥ 0.32 subgroup (3.71 ± 1.01) was significantly higher than in the FLT3-ITD MR < 0.32 (2.35 ± 0.39) and FLT3-wt (1.14 ± 0.21) subgroups (P ≤ 0.0001, respectively, Fig. 2C). In addition, a significantly higher level of the CXCR4 mRNA in the FLT3-ITD MR < 0.32 subgroup was detected than in the FLT3-wt subgroup (1.14 ± 0.21, P = 0.001, Fig. 2C). The level of the Pim-1 mRNA in the FLT3-ITD MR ≥ 0.32 subgroup (2.79 ± 0.94) was significantly higher than in the FLT3-ITD MR < 0.32 (1.77 ± 0.11) and FLT3-wt (1.18 ± 0.24) subgroups (P ≤ 0.0001, respectively, Fig. 2D). However, significantly higher level of the Pim-1 mRNA in the FLT3-ITD MR < 0.32 subgroup was detected than in the FLT3-wt subgroup (1.18 ± 0.24, P = 0.018, Fig. 2D).

### Changes in the proliferation, apoptosis, and chemotaxis of leukemia cells treated with different concentrations of a CXCR4 antagonist (AMD3100) and/or sorafenib

**Figure 3:**
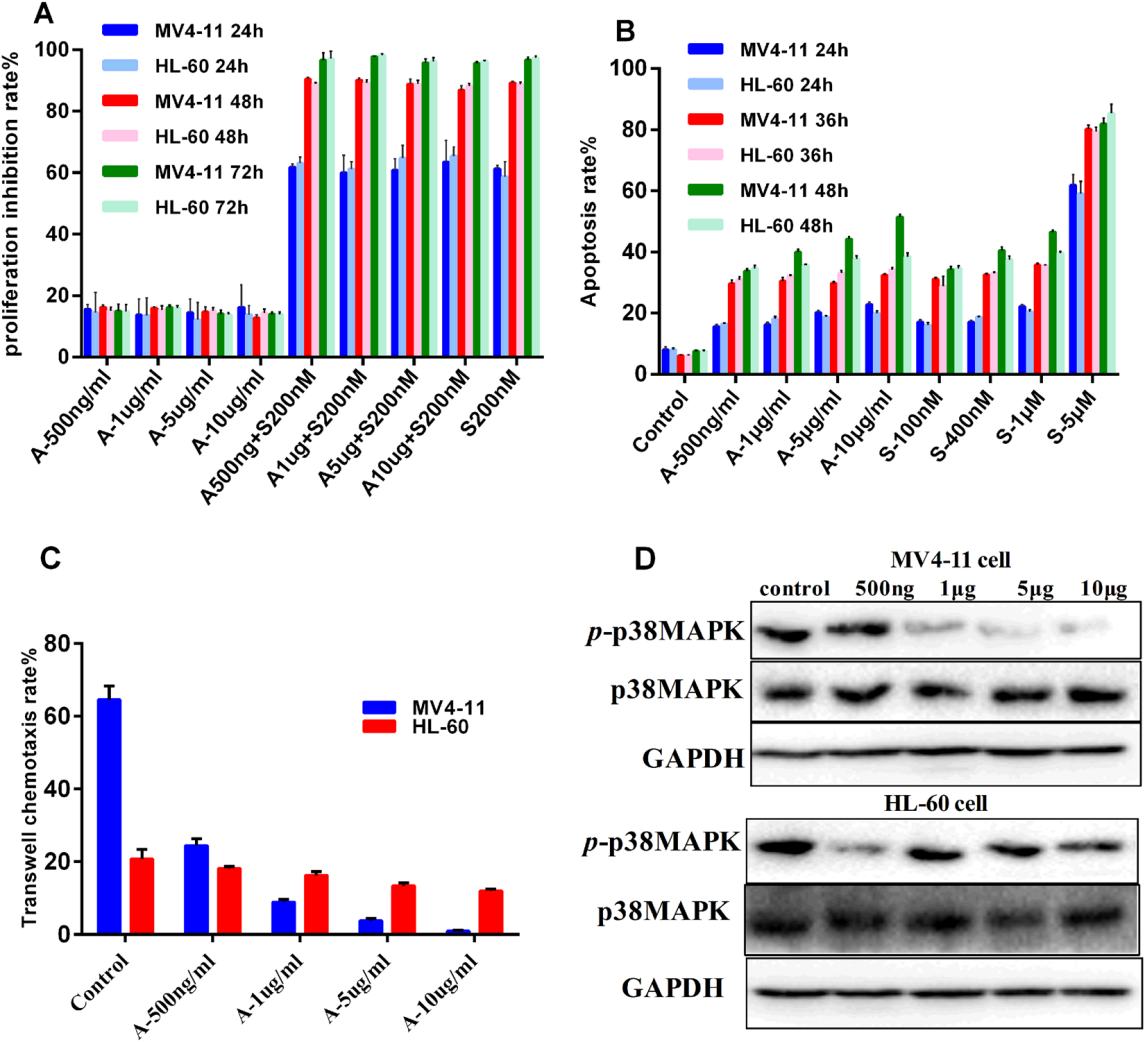
The effect of different concentrations of the CXCR4 antagonist AMD3100 and/or sorafenib on AML cell proliferation inhibition, apoptosis and chemotaxis. (**A**–**C**) The median value for each group is represented by the height of the bar in the histogram, and the error bars are shown as a “T” above each bar in the histogram. (**A**) Effect of different concentrations of AMD3100 and/or sorafenib on the inhibition of AML cell proliferation. A significant difference in the inhibition rate of proliferation of MV4-11 and HL-60 cells treated with the optimal therapeutic concentration of sorafenib (200-nM) was not observed (P > 0.05). Compared with sorafenib alone, the combination of AMD3100 and sorafenib(200 nM) did not increase the proliferation inhibition rate of MV4-11 cells expressing FLT3-ITD mutations. The inhibitory effects of AMD3100 on proliferation did not differ in MV4-11 cells and HL-60 cells (P > 0.05); AMD3100 alone was inferior to both sorafenib alone and the combination of sorafenib and AMD3100 (P < 0.05). (**B**) Effect of different concentrations of AMD3100 and sorafenib on AML cell apoptosis. The apoptosis rates of MV4-11 cells and HL-60 cells treated with different doses of sorafenib and AMD3100 were significantly higher than the negative control groups after 24, 36, and 48 h of incubation (P < 0.05). AMD3100 induced apoptosis in MV4-11 cells in a concentration- and time-dependent manner. The apoptosis rates in the group treated with a high concentration of sorafenib (5 μM) were significantly higher than the groups treated with AMD3100 at all observed time points (P < 0.05). Thus, AMD3100 induced a low level of apoptosis in leukemia cells, but this effect was not very pronounced. (**C**) The chemotaxis rate of MV4-11 and HL-60 cells toward CXCL12 was inhibited after an incubation with different concentrations of AMD3100 (500 ng/ml, 1 μg/ml, 5 μg/ml, and 10 μg/ml) for 4 hours. The chemotaxis rate of MV4-11 cells was higher than HL-60 cells treated with 500 ng/ml AMD3100; however, the chemotaxis rates of MV4-11 cells treated with 1, 5, or 10 μg/ml AMD3100 were significantly lower than HL-60 cells in each corresponding AMD3100 concentration group (all P < 0.05). (**D**) The levels of the p-p38 MAPK/p38 MAPK proteins expression in HL-60 and MV4-11 cells in subject to the chemotaxis assay were altered by different concentrations of AMD3100 (0, 500 ng/ml, 1 μg/ml, 5 μg/ml, and 10 μg/ml). AMD3100 decreased the level of the p-p38 MAPK (Thr180/Tyr182) protein in MV4-11 cells in a concentration-dependent manner, but did not appreciably change the p-p38 MAPK level in HL-60 cells.

First, we investigated the effectiveness and the optimal therapeutic concentration of sorafenib and determined it to be 200 nM. We thus selected this concentration to administer in combination with AMD3100 in the proliferation inhibition experiment. Compared with sorafenib alone, the combination of AMD3100 and sorafenib did not increase the proliferation inhibition rate of MV4-11 cells expressing FLT3-ITD mutants (Fig. 3A). Moreover, the inhibitory effects of AMD3100 on the proliferation of MV4-11 cells and HL-60 cells did not differ; AMD3100 alone was inferior to both sorafenib alone and the combination of sorafenib and AMD3100 (P < 0.05, Fig. 3A).

The results of the flow cytometry apoptosis analysis revealed significantly higher apoptosis rates of MV4-11 cells and HL-60 cells treated with different doses of sorafenib and AMD3100 than the negative control groups after 24, 36, and 48 h of incubation (P < 0.05). AMD3100 induced apoptosis in MV4-11 cells in a concentration- and time-dependent manner. (details are shown in Fig. 3B and the Supplementary Data). The apoptosis rates in the group treated with a high concentration of sorafenib (5 μM) were significantly higher than in the groups treated with AMD3100 at all observed time points (P < 0.05, Fig. 3B). Thus, AMD3100 induced a low level of apoptosis in leukemia cells, but this effect was not very pronounced (Fig. 3B).

Based on the results of the Transwell assay, the ability of MV4-11 cells overexpressing CXCR4 to undergo chemotaxis toward CXCL12 was significantly increased compared with HL-60 cells expressing low levels of CXCR4 in the control group (64.57 ± 3.72 vs. 20.76 ± 2.70, P = 0.001, Fig. 3C). In the AMD3100 group, the chemotaxis rates of MV4-11 cells toward CXCL12 (SDF-1α) were 24.44 ± 1.90 (500 ng/ml AMD3100), 8.90 ± 0.81 (1 μg/ml AMD3100), 3.74 ± 0.72 (5 μg/ml AMD3100) and 0.89 ± 0.31 (10 μg/ml AMD3100, details are shown in the Supplementary Data). Thus, AMD3100 blocked the chemotaxis of MV4-11cells toward CXCL12 in a concentration- dependent manner, and chemotaxis was almost completely blocked in the presence of 10 μg/ml AMD3100 (P < 0.05). The chemotaxis rates (toward CXCL12) of HL-60 cells without FLT3-ITD mutations were 18.14 ± 0.53 (500 ng/ml AMD3100), 16.20 ± 1.08 (1 μg/ml AMD3100), 13.34 ± 0.81 (5 μg/ml AMD3100) and 11.99 ± 0.49 (10 μg/ml AMD3100), indicating a decreasing tendency of leukemia cells to undergo chemotaxis toward CXCL12 with increasing AMD3100 concentrations (P > 0.05). The chemotaxis rate of MV4-11 cells was higher than HL-60 cells treated with for 500 ng/ml AMD3100; however, the chemotaxis rates of MV4-11 cells treated with 1 μg/ml, 5 μg/ml, or 10 μg/ml AMD3100 were significantly lower than HL-60 cells in each corresponding AMD3100 concentration group (all P < 0.05, Fig. 3C). In general, AMD3100 significantly inhibited the chemotaxis of MV4-11 cells toward CXCL12 compared to HL-60 cells (P < 0.05, Fig. 3C).

In addition, the change in p-p38 mitogen-activated protein kinase (MAPK)/p38 (Thr180/Tyr182) levels in MV4-11 and HL-60 cells after an incubation with different concentrations of the CXCR4 antagonist AMD3100 (500 ng/ml, 1 μg/ml, 5 μg/ml, and 10 μg/ml) was observed. AMD3100 decreased the levels of the p-p38 MAPK (Thr180/Tyr182) protein in MV4-11 cells in a concentration-dependent manner, but did not appreciably change the levels of the p-p38 MAPK protein in HL-60 cells (Fig. 3D).

## Discussion

The FLT3 ligand and its receptor FLT3 have been implicated in the survival, proliferation, adhesion and maintenance of human CD34+/CD38− progenitor cells^13,21^. FLT-ITDs, the main forms of FLT3 mutations, are present in approximately 25% of adult patients with AML and are associated with an extremely poor prognosis^22,23^. Although several FLT3-ITD antagonists have been developed and investigated either as single agents or in combination with chemotherapy in patients with AML carrying FLT3-ITD mutations, the successful clinical use of FLT3-ITD antagonists has been challenged by the development of drug resistance and limited clinical efficacy^8,24^. The mechanism responsible for the resistance of FLT3-ITD-expressing AML cells to FLT3-ITD antagonists is multifactorial and includes components such as additional mutations other than preexisting FLT3-ITD mutations and microenvironment-mediated resistance^9,25^. According to some studies, FLT3-ITD mutations enhance leukemia cell chemotaxis toward CXCL12, thus providing a drug resistance mechanism underlying the poor effect of FLT3-ITD antagonists^14,26^. Based on these data, FLT3-ITD facilitates the interaction between leukemia cells and the microenvironment by enhancing CXCL12/CXCR4 signaling. However, the mechanisms by which FLT3-ITDs regulate the CXCL12/CXCR4 axis are unknown. We aim to clarify the effect of FLT3-ITD mutations on CXCR4 expression in AML.

In the present study, we analyzed the expression of FLT3-ITD mutations and CXCR4 in 466 patients with *de novo* AML. The overall prevalence of FLT3-ITD mutations was 21.67% (101 of 466), consistent with other reports^5,7^. Among the included patients, a signifcantly increase was observed in the number of patients with AML FAB subtype M5 who carried FLT3-ITD mutations, 35.82% of these patients carried an FLT3-ITD mutation. In contrast, FAB subtypes M2 (15.61%) and M6 (7.41%) were significantly less frequently associated with FLT3-ITD mutations,which is also consistent with the report that the stimulation of hematopoietic progenitors with FLT3, but not other growth factors, promotes monocyte differentiation^27^. In the present study, the FLT3-ITD mutation rate was significantly higher in patients with monocytic leukemia, who have a poorer prognosis, than in patients with granulocytic leukemia. Then, patients with FAB subtypes M4 and M5 harboring monocytic leukemia cells exhibited significantly higher CXCR4 expression than patients with other FAB subtypes, consistent with the characteristics of peripheral blood hyperleukocytosis, skin damage, hepatosplenomegaly, extramedullary infiltration and a poor prognosis in patients with monocytic leukemia. Indeed, CXCR4 overexpression is associated with extramedullary infiltration in many hematological malignancies^28,29^, and our study indicated that the FLT3-ITD MR was positively correlated with the RFI for CXCR4 expression on leukemia cells. Many studies have verified that a high FLT3-ITD MR is associated with a poor prognosis for patients with AML^6,7^. By binding to its ligand CXCL12 that is secreted from stromal cells, CXCR4 signals through the CXCL12/CXCR4 axis and plays important roles in the migration, proliferation and apoptosis of AML cells. Leukemia cells with high CXCR4 expression are recruited to and reside in the bone marrow niche, where they protect leukemia cells from cytotoxic chemotherapeutics and represent a reservoir for minimal residual disease and relapses, thus leading to chemotherapy resistance and disease relapse in patients with AML^11,12^. Therefore, we speculated that FLT3-ITD mutations potentiallyupregulate CXCR4 expression on the surface of leukemia cells and enhance the chemotaxis of AML cells toward CXCL12, consistent with previously reported results^14,26,30^.

The mechanism by which FLT3-ITD mutations increase CXCR4 expression in AML remains unclear. FLT3-ITD mutations allow the ligand-independent activation and phosphorylation of the FLT3 receptor, leading to the aberrant activation of multiple downstream pathways, such as the phosphatidylinositol 3-kinase (PI3K)/AKT and MAPK/extracellular signal-regulated kinase (ERK) pathways^31^. In contrast to wild-type FLT3 signaling, FLT3-ITD mutations potently activate the STAT5 pathway^15^. The aberrant activation of the STAT or Janus kinase (JAK)/STAT pathways is recognized as a common characteristic of several hematopoietic malignancies^32^. STAT5 induces the expression of its target genes, such as, cyclin D1, c-myc and the protooncogene Pim-1, which mediates the proliferative and antiapoptotic behavior of leukemia cells via the FLT3-ITD signaling pathway^33,34^. Among the STAT5 target genes, Pim-1, an oncogenic serine/threonine kinase, was identified as one of the first known target genes of STAT5 and is upregulated by STAT5^16,35,36^. In addition, Pim-1 is essential for the surface expression and intracellular processing of CXCR4^17,37^. We compared the differences in p-STAT5/STAT5, Pim-1 and CXCR4 levels between groups of patients with AML carrying FLT3-ITD mutations and wild-type FLT3-wt. Consistent with the aforementioned reports, levels of the p-STAT5, Pim-1 and CXCR4 proteins were significantly increased with an increase in the FLT3-ITD MR in the present study. Furthermore, the expression of the CXCR4 and Pim-1 mRNAs was significantly increased with an increase in the FLT3-ITD MR. Our data indirectly provided evidence implicating a role for FLT3-ITD gene mutations in modulating the CXCL12/CXCR4 axis by increasing the p-STAT5 level, which, in turn, upregulated the expression of the target gene Pim-1. Therefore, the poor prognosis patients with AML carrying FLT3-ITD- mutations might result from increased CXCR4 expression. However, the elucidation of its mechanism requires further research in the future.

Next, we briefly verified the effect of the CXCR4 antagonist AMD3100 on blocking signaling via the CXCL12/CXCR4 axis by assessing the proliferation, apoptosis and chemotaxis of AML cells with or without FLT3-ITD mutations. No differences in the inhibition of proliferation and the induction of apoptosis were observed between FLT3-ITD-mutated MV4-11 cells and FLT3-wt HL-60 cells treated with AMD3100. Notably, AMD3100 significantly inhibited the chemotaxis of FLT3-ITD-mutated MV4-11 cells toward CXCL12 compared to FLT3-wt HL-60 cells (P < 0.05); chemotaxis was almost completely blocked by 10 μg/ml AMD3100. Recently, the CXCL12/CXCR4 axis was reported to function through p38 MAPK signaling to drive the progression and metastasis of various cancers, including follicular lymphoma and lung, thyroid, colorectal and breast carcinomas^38,39^. In addition, CXCL12 induces p38 MAPK phosphorylation in pancreatic cancer^40^. The p38 MAPK protein is a serine/threonine-directed kinase that is classified as a “stress-activated” kinase in the MAPK family that, in concert with various signaling cascades such as the JNK, ERK, AMPK and PI3K pathways, regulate the balance between cell survival and cell death, with direct effects on the development of various cancers^38^. Consistent with the results of these studies, AMD3100 decreased the level of p-p38 MAPK (Thr180/Tyr182) in MV4-11 cells, but not in FLT3-wt HL-60 cells, in the present study.

Our study confirms that FLT3-ITD mutations apparently increase the levels of p-STAT5 and subsequently upregulate the expression of the target gene Pim-1, leading to an increase in the expression of CXCR4 on the surface of leukemia cells that in turn contributes to chemotherapy resistance and disease relapse. A decrease in p-p38 MAPK levels may be the mechanism by which CXCR4 antagonists disrupt the chemotaxis of FLT3-ITD-expressing AML cells toward CXCL12. Thus, the combination of CXCR4 antagonists with FLT3 inhibitors may improve the sensitivity of AML cells to chemotherapy and overcome drug resistance.

### Ethics statement

The study protocol was reviewed and approved by the Ethics Committee of West China Hospital of Sichuan University. All biological samples were obtained from patients and controls that had provided written informed consent in accordance with the tenets of the Declaration of Helsinki.

## Supplementary information

supplemental data
