# Supplementary material for: The FLT3-ITD mutation and the expression of its downstream signaling intermediates STAT5 and Pim-1 are positively correlated with CXCR4 expression in patients with acute myeloid leukemia
Source: Sci Rep. 2019 Aug 21;9:12209. doi: 10.1038/s41598-019-48687-z (PMC6704161; doi:10.1038/s41598-019-48687-z)
Supplement: Supplementary file 1 — supplemental data [file 41598_2019_48687_MOESM1_ESM.docx]

**The FLT3-ITD mutation and the expression of its downstream signaling intermediates STAT5 and Pim-1 are positively correlated with CXCR4 expression in patients with acute myeloid leukemia**

# Tingyong Cao^1#^, Nenggang Jiang^2#^, Hongyan Liao^2^, Xiao Shuai^1^, Jun Su^2^, Qin Zheng^2*^

1. Department of Hematology, West China Hospital of Sichuan University, Chengdu 610041, China
2. Department of Laboratory Medicine, West China Hospital of Sichuan University, Chengdu 610041, China

*Corresponding Author: Qin Zheng, E-mail: [zhengqinhx@scu.edu.cn](mailto:zhengqinhx@scu.edu.cn)

^#^ These authors contributed equally to this work.

1. **Apoptosis assay of MV4-11 cells incubated with different concentrations of AMD3100 (0, 500 ng/ml, 1 μg/ml, 5 μg/ml, and 10 μg/ml) for 24 h.**


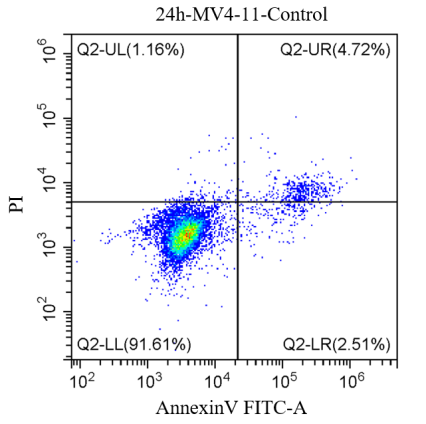

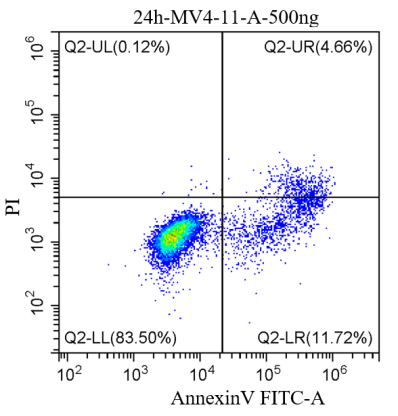

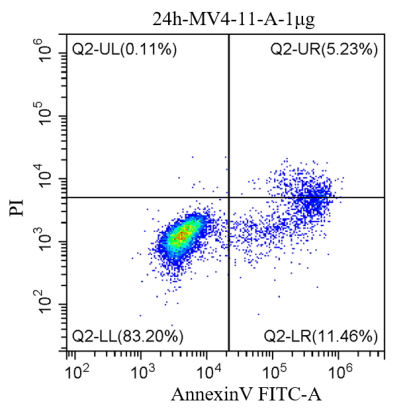

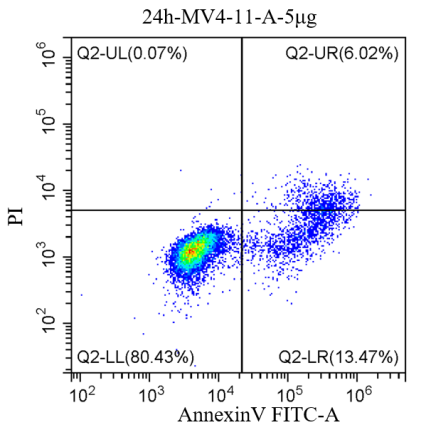

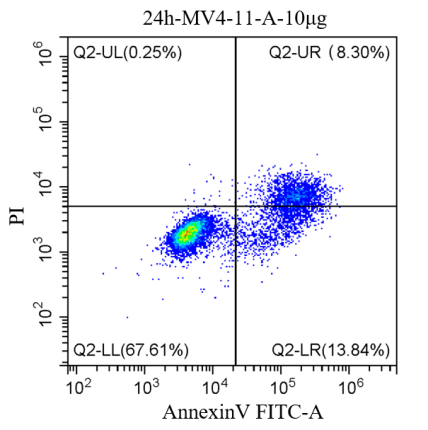


**2.MV4-11 cells incubated with different concentrations of AMD3100 (0, 500 ng/ml, 1 μg/ml, 5 μg/ml, and 10 μg/ml) for 4 hours migrated to the lower chamber of the Transwell plates.**

**
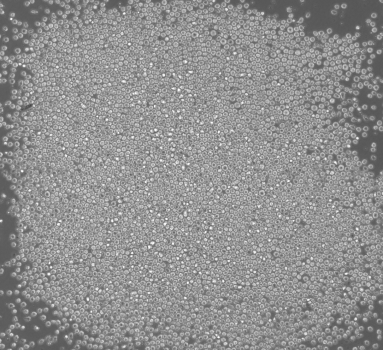

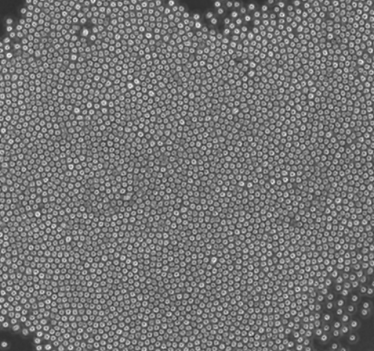

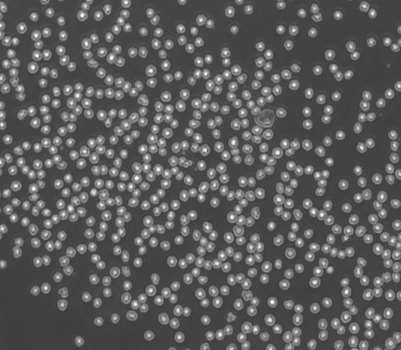

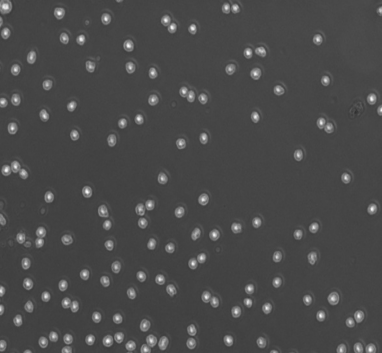

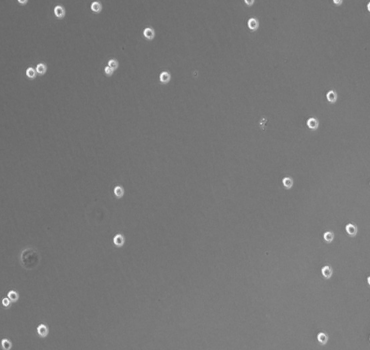
**

**4h-MV-11-A-1ug**

**4h-MV-11-A-10ug**

**4h-MV-11-A-5ug**

**4h-MV-11-A-500ng**

**4h-MV-11-control**
